# Acarbose Reduces Blood Glucose by Activating miR-10a-5p and miR-664 in Diabetic Rats

**DOI:** 10.1371/journal.pone.0079697

**Published:** 2013-11-18

**Authors:** Qian Zhang, Xinhua Xiao, Ming Li, Wenhui Li, Miao Yu, Huabing Zhang, Zhixin Wang, Hongding Xiang

**Affiliations:** Key Laboratory of Endocrinology, Ministry of Health, Department of Endocrinology, Peking Union Medical College Hospital, Peking Union Medical College, Chinese Academy of Medical Sciences, Beijing, China; Dasman Diabetes Institute, Kuwait

## Abstract

MicroRNAs (miRNAs) are non-coding RNA molecules involved in the post-transcriptional regulation of a large number of genes, including those involved in glucose metabolism. Acarbose is an α-glucosidase inhibitor that improves glycemic control by decreasing the intestinal absorption of glucose, thereby decreasing the elevation of postprandial blood glucose. However, acarbose is poorly absorbed into the blood stream from the gut. Therefore, the exact mechanisms by which acarbose affects glucose metabolism are unclear. This study investigated the effect of acarbose on glucose metabolism in diabetic rats and tested the hypothesis that acarbose acts directly through miRNA-regulated expression in the intestinal epithelium. Rats were divided into four groups: a control group, a diabetic group (DM), a low dose of acarbose group (AcarL) and a high dose of acarbose group (AcarH). Ileum samples were analyzed using miRCURY LNA™ microRNA Array, qPCR and immunohistochemistry. We found that 8-week treatment with acarbose significantly decreased fasting blood glucose. Oral glucose tolerance tests (OGTT) showed that blood glucose was significantly reduced in the AcarL and AcarH groups at 30 min, 60 min and 120 min after oral glucose administration. We found that miR-151*, miR-10a-5p, miR-205, miR-17-5p, miR-145 and miR-664 were up-regulated in the AcarH group, while miR-541 and miR-135b were down-regulated. Through target gene analysis, real time PCR and immunohistochemistry verification, we found that these miRNAs suppressed the expression of proinflammatory cytokines [IL6 (interleukin 6) and TNF (tumor necrosis factor)] and mitogen activated protein kinase 1 (MAPK1). Our data suggest that acarbose can improve blood glucose in diabetic rats through the MAPK pathway and can down-regulate proinflammatory factors by activating miR-10a-5p and miR-664 in the ileum.

## Introduction

Diabetes mellitus is one of the most common chronic diseases worldwide, and continues to increase in incidence and significance, as changing lifestyles lead to reduced physical activity, and increased obesity. Type 2 diabetes mellitus is an emerging worldwide health problem, with the number of global cases of type 2 diabetes projected to double to 350 million by the year 2030 [1]. Diabetes is an independent risk factor for cardiovascular disease [2], [3] and is the leading cause of morbidity and mortality in the developed world [4]–[6].

Acarbose is an α-glucosidase inhibitor that delays the digestion of complex carbohydrates and disaccharides to absorbable monosaccharides by reversibly inhibiting α-glucosidases within the intestinal brush border, thereby attenuating postprandial blood glucose peaks [7]. Clinical trials have demonstrated that acarbose generally improves glycemic control in patients with diabetes mellitus that can be managed by diet alone, or in combination with other antidiabetic therapies, as evidenced by decreased postprandial plasma glucose and glycosylated hemoglobin. It does not appear to directly alter insulin resistance, but it may lower postprandial plasma insulin levels. However, the bioavailability of acarbose is low [8], which is attributed to its poor aqueous solubility.

MicroRNAs (miRNAs) are short (21–23 nucleotides), endogenous, non-coding RNA molecules. miRNAs regulate gene expression by imperfect base pairing with the 3′-untranslated regions of mRNAs, resulting in mRNA decay or translational repression [9]. miRNAs have distinct spatial and temporal expression patterns in cells and tissues and regulate several processes, including hematopoiesis, development, cell differentiation, proliferation and apoptosis [10], [11]. They are implicated in several diseases, including diabetes.

We therefore hypothesized that acarbose directly alters the intestinal expression of miRNAs to regulate glucose metabolism. To provide molecular evidence for this mechanism, we used a rat model of type 2 diabetes to investigate differential miRNA expression in rat intestines after treatment with acarbose.

## Materials and Methods

### 1. Animal Models, Grouping, and Treatment

Male Sprague-Dawley rats (280–320 g) were purchased from the Institute of Laboratory Animal Science, Chinese Academy of Medical Sciences and Peking Union Medical College (Beijing, China, SCXK-2012-0007). As previously described [12], diabetic rats were fed a high-fat diet (40% of calories as fat) for 4 weeks, and then were administered a single dose of streptozotocin (STZ, 50 mg/kg, tail vein) formulated in 0.1 mmol/L citrate buffer, pH 4.5 (Sigma–Aldrich, MO, USA). One week after the STZ injection, the random blood glucose level of the diabetic rats was measured to confirm hyperglycemia. Random blood glucose above 16.7 mmol/L was used to define rats as diabetic. Diabetic rats were fed a high-fat diet throughout the experiment. Diabetic rats with a similar degree of hyperglycemia were randomly divided into three groups: vehicle, low dose acarbose (AcarL), and high dose acarbose (AcarH) groups (n = 10, in each group). The typical human daily dose of acarbose is 300 mg/60 kg body weight. According to the formula: d_rat_ = d_human_ × 0.71/0.11 [13], the corresponding dose of acarbose for rats is 32.28 mg/kg per day. Therefore, we selected 30 and 60 mg/kg per day as low and high dosages, respectively. The control (n = 10) and the diabetic group received 0.5% saline, whereas the AcarL and AcarH groups were given acarbose (Bayer Health Care Co., Germany) at doses of 30 and 60 mg/kg in a 0.5% saline solution, respectively. The drug was administered once daily for 8 weeks using a gastric gavage. All animals were housed in an environmentally controlled room at 25°C with a 12 h light-dark cycles and were given free access to food and water throughout the experimental period. Fasting animals were allowed free access to water. After 6 weeks of treatment, an oral glucose tolerance test (OGTT) was performed. After 8 weeks of treatment, blood samples were taken from rats after anesthesia. The rats were then sacrificed. Some terminal ileums were collected for performing the microarray and quantitative real-time reverse transcription PCR (qRT-PCR) analysis. Other terminal ileums were fixed in 10% neutralized formalin for immunohistochemical staining. All procedures involving animals were approved by the animal care and use committee of the Peking Union Medical College Hospital (Beijing, China, MC-07-6004) and were conducted in compliance with the Guide for the Care and Use of Laboratory Animals, 8th ed., 2011. All surgeries were performed under sodium pentobarbital anesthesia, and all efforts were made to minimize suffering.

### 2. Measurement of Body Weight and Fasting Blood Glucose

Body weight was measured every 2 weeks. The 6-h fasting blood glucose (FBG) level was measured every 2 weeks using a Contour TS glucometer (Bayer) with blood from a tail bleed.

### 3. Oral Glucose Tolerance Test (OGTT)

After the rats had fasted for 6 h, 2.2 g/kg of glucose was orally administered. Then, blood samples were collected from tail veins at 0 (prior to the glucose load), 30, 60 and 120 min (after the glucose load) for a glucose assay. The area under the curve (AUC) was calculated for blood glucose (BG) during the OGTT: AUC = 0.5×(BG0+ BG30)/2+ (BG30+BG60)/2+1×(BG60+BG120)/2.

### 4. Serum IL6 and TNF-α Level Analysis

At week 8, blood samples were collected after euthanasia and centrifuged at 1000 g for 10 min. Serum was stored in aliquots at −80°C to assay serum interleukin 6 (IL6) and tumor necrosis factor α(TNF-α). Serum IL6 and TNF-α levels were measured by enzyme-linked immunosorbent assay (ELISA, Abcam, UK).

### 5. miRCURY LNA™ microRNA Array Experiment

The miRCURY LNA™ miRNA array system contains 3100 capture probes, covering all rat microRNAs (388 miRNAs) that have been annotated in miRBase 18.0 as well as all viral microRNAs associated with rats. Total RNA from the iliem of AcarH group and DM group was harvested using TRIzol (Invitrogen) and an miRNeasy Mini Kit (QIAGEN) according to the manufacturers’ instructions. After the RNA was quantified using a NanoDrop 1000, the samples were labeled using a miRCURY™ Hy3™/Hy5™ Power labeling kit and hybridized on a miRCURY™ LNA Array v.18.0 (Exiqon). Following washing, the slides were scanned using an Axon GenePix 4000B microarray scanner.

### 6. Gene Array Data Analysis

Normalization was performed with a per-chip 50^th^ percentile method that normalizes each chip on its median, allowing for comparison among chips.

### 7. miRNA Target Gene Prediction

miRNA target genes were identified using the miRWalk online database (http://www.umm.umi-heidelberg.de/apps/zmf/mirwalk/). miRWalk provides information on published pathway targets from the Kyoto Encyclopedia of Genes and Genomes (KEGG, http://www.genome.jp/kegg/). The gene functions were obtained from NCBI-Gene (http://www.ncbi.nlm.nih.gov).

### 8. miRNA Quantitative Real-time PCR (qRT-PCR)

Total RNA (5 ng) was reverse-transcribed using a Taqman™ MicroRNA Reverse Transcription kit (Applied Biosystems) and the miRNA-specific reverse-transcription primers provided with TaqMan™ MicroRNA Assays (Applied Biosystems). For reverse-transcription, a PTC-225 Peltier Thermal Cycler (MJ Research Inc., Waltham, Massachusetts) was used. The reaction conditions were 16°C for 30 min, 42°C for 30 min and 85°C for 5 min. The generated miRNA-specific cDNA was amplified using a TaqMan™ Universal PCR master mix II (Applied Biosystems) and the respective specific probe provided with TaqMan™ Small RNA Assays (Applied Biosystems). PCR was performed using a CFX-96TOUCH (Bio-Rad) detection system. Amplification was performed at 95°C for 10 min, followed by 40 cycles of 95°C for 15 sec and 60°C for 60 sec. U6 small nuclear RNA was used as an endogenous control. The fold change in miRNA level was calculated by the equation: fold change = 2^−△△Ct^, where △Ct = Ct_miRNA_-Ct_U6_ and △△Ct = △Ct_acarbose treated samples_− △Ct_untreated diabetic samples_ [14].

### 9. Target Gene Validation by qRT-PCR

For the validation of miRNA target genes, qRT-PCR analyses of RNAs were performed using SYBR Green. Each qRT-PCR assay was repeated using three biological replicates, and each analysis consisted of three technical replicates. Before PCR analysis, each sample of total RNA was treated with RNase-free DNase (Qiagen, Valencia, CA, USA). RNA was reverse-transcribed by Superscript II (Invitrogen, CA, USA). The primers were designed using the Applied Biosystems (Foster City, CA, USA) Primer Express™ design software. Primers were purchased from Applied Biosystems (Table 1). Using the ABI Prism 7700 Sequence Detection System, the following reaction conditions were used: an initial denaturation at 48°C for 30 min, followed by 95°C for 15 min, and then 40 cycles of 95°C for 15 sec, and 55°C for 1 min, and a final unlimited 4°C hold. The sequences of the primers are listed in Table 1. The signal of the housekeeping gene *Gapdh* (glyceraldehyde-3-phosphate dehydrogenase) was used for normalization. The relative quantification of the mRNA between the acarbose treated groups and DM rats was calculated using the comparative Ct method.

**Table 1:** Olgonucleotide sequences for mRNA Q-PCR.

Gene symbol	Forward primer	Reverse primer
*Il6*	GACAACTTTGGCATTGTGG	ATGCAGGGATGATGTTCTG
*MAPK1*	ATGGCGGCGGCGGCGGCGGCGGGCGCGGGC	TTAAGATCTGTATCCTGGCTGGAATCTAGC
*Tnf*	CTTGTCTACTCCCAGGTTCTCTTC	TAAGTACTTGGGCAGATTGACCT
*Gadph*	GACCCCTTCATTGACCTCAAC	CGCTCCTGGAAGATGGTGATG

*Il6*: interleukin 6; *Mapk1*: mitogen activated protein kinase 1; *Tnf*: tumor necrosis factor; *Gadph*: glyceraldehyde-3-phosphate dehydrogenase.

### 10. Immunohistochemical Staining

Ileum from the AcarH and DM groups (n = 6 in each group) were fixed in 10% neutral buffered formalin, cast in paraffin, sliced into 4-µm sections and placed onto microscope slides. After the removal of the paraffin by xylene and dehydration by graded alcohol, the slides were immersed into distilled water. Ileum sections were then transferred into a 10-mmol/L citrate buffer solution (pH 6.0) and heated at 80°C for 5 min for antigen retrieval. After washing, 3.0% peroxide was applied for 20 min to block the activity of endogenous peroxidase. To avoid nonspecific staining, the sections were incubated in blocking solution (5% BSA) for 1 h at room temperature, followed by treatment with mouse monoclonal anti-TNF-α antibody (1∶50, Abcam Inc., UK), rabbit polyclonal anti-IL6 antibody (1∶100, Abcam lnc., UK), or rabbit polyclonal anti-MAPK1 antibody (1∶100, Abcam Inc., UK) where indicated, overnight at 4°C. Negative control sections were stained under identical conditions by substituting the primary antibody with equivalent concentrations of normal rabbit IgG. After washing with phosphate-buffered saline, the slides were incubated with a labeled streptavidin biotin reagent, following the manufacturer’s instructions. Immunoreactive products were visualized with the DAB reaction. Sections were counterstained with hematoxylin for 15 sec. Brownish yellow granular or linear deposits were interpreted as positive areas. Three observers blinded to the clinical information evaluated the immunohistochemical staining scores independently. Staining intensity was graded semi-quantitatively using the H-SCORE [15] which was calculated using the following equation: H-SCORE = ∑Pi (i +1), where i is the intensity of staining with a value of 1, 2 or 3 (mild, moderate, or strong, respectively) and Pi is the percentage of epithelial cells stained with different intensity, varying from 0% to 100%. The results are expressed as the mean ± SE. Differences between the groups were statistically analyzed with a one-way analsis of variance (ANOVA). A *P* value of <0.05 was considered significant.

### 11. Statistical Analysis

All results are expressed as the mean ± standard deviation (SD). Statistical analyses were performed with ANOVA followed by Student’s *t* test. *P*<0.05 was considered statistically significant. Analyses were performed with SPSS 11.0 (SPSS, Inc., Chicago, IL, USA).

## Results

### 1. Acarbose had No Effect on the Body Weight of DM Rats

**Figure 1:**
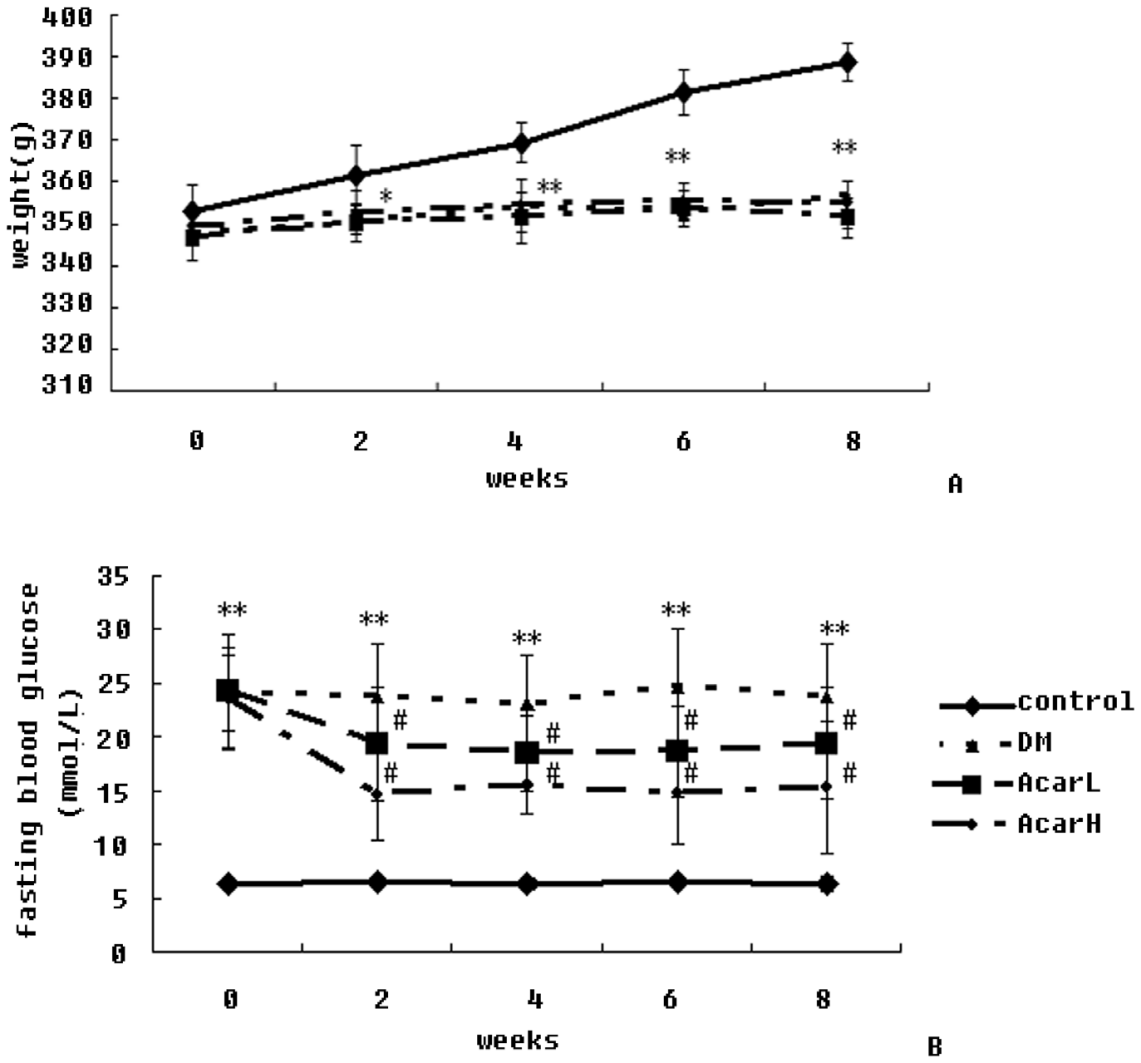
Body weight (A) and fasting blood glucose (B) before and after acarbose treatment in rats. Data represent mean ± SD (n = 10 per group). **P<0.01 versus the control group; ^#^ *P*<0.05 versus DM group.

The mean body weight of diabetic rats significantly decreased compared to the control rats at week 2 (*P*<0.05), week 4 (*P*<0.01), week 6 (*P*<0.01) and week 8 (*P*<0.01). No significant differences were noted between the DM group and the acarbose-treated groups (Figure 1A).

### 2. Acarbose Decreased the Fasting Blood Glucose of DM Rats

The fasting blood glucose (FBG) levels of diabetic rats were significantly higher than those of control rats at week 0 (*P*<0.01), week 2 (*P*<0.01), week 4 (*P*<0.01), week 6 (*P*<0.01) and week 8 (*P*<0.01). FBG in the acarbose-treated group decreased significantly at week 2 (*P*<0.05), week 4 (*P*<0.05), week 6 (*P*<0.05), and week 8 (*P*<0.05) compared to the DM group (Figure 1B).

### 3. Acarbose Regulated the Glucose Tolerance of DM Rats

**Figure 2:**
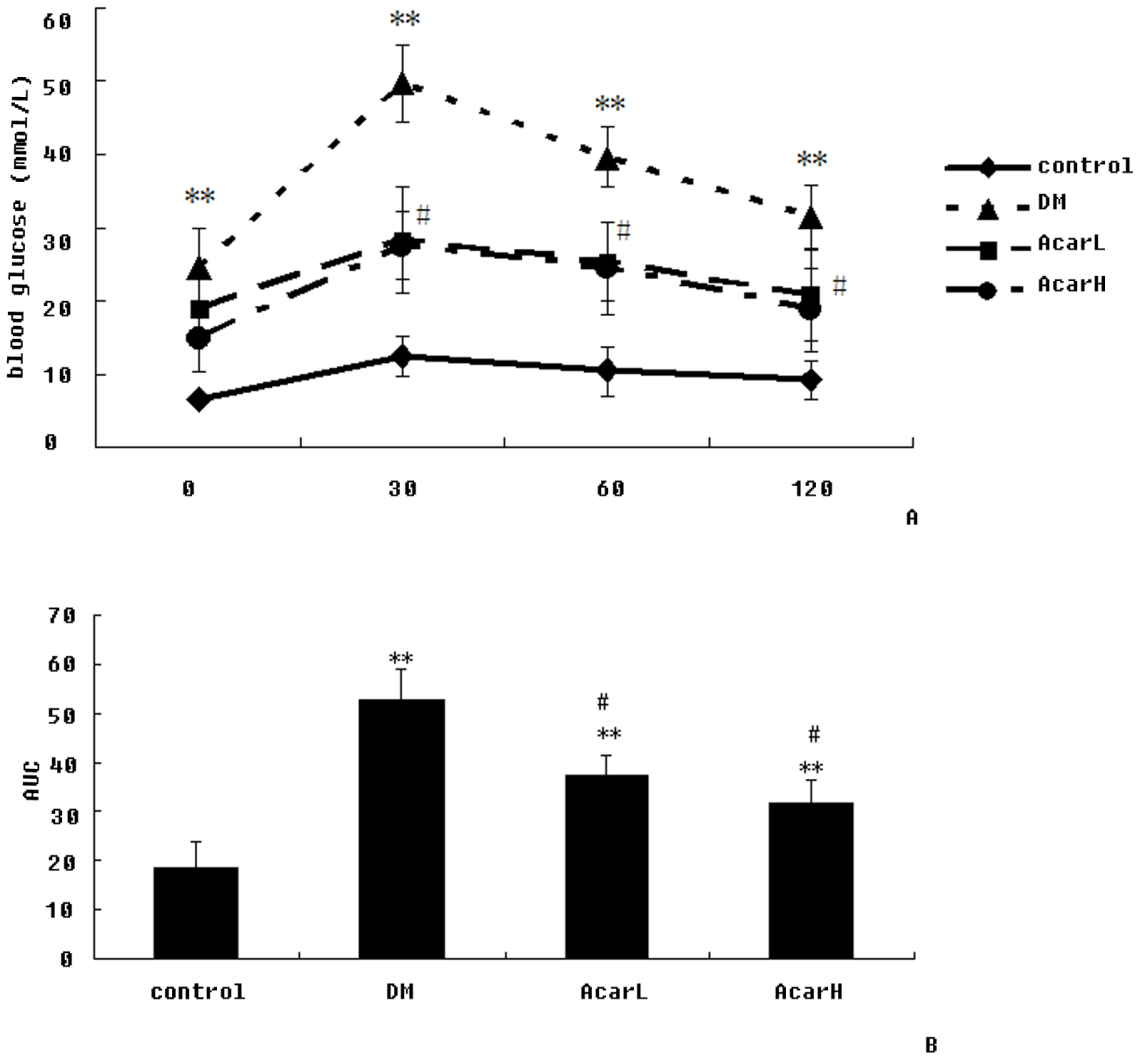
The effect of acarbose on oral glucose tolerance test blood glucose (A) and AUC (B) in rats. Data represent mean ± SD (n = 10 per group). **P<0.01 versus the control group; ^#^ *P*<0.05 versus DM group.

The blood glucose levels of the DM group were higher than those of the control group before and at 30 min (*P*<0.01), 60 min (*P*<0.01) and 120 min (*P*<0.01) after oral glucose administration. Blood glucose levels of the acarbose-treated groups significantly decreased at 30 min, 60 min and 120 min after oral glucose administration (*P*<0.05, Figure 2).

### 4. Acarbose Reduced Serum IL6 and TNF-α in DM Rats

**Figure 3:**
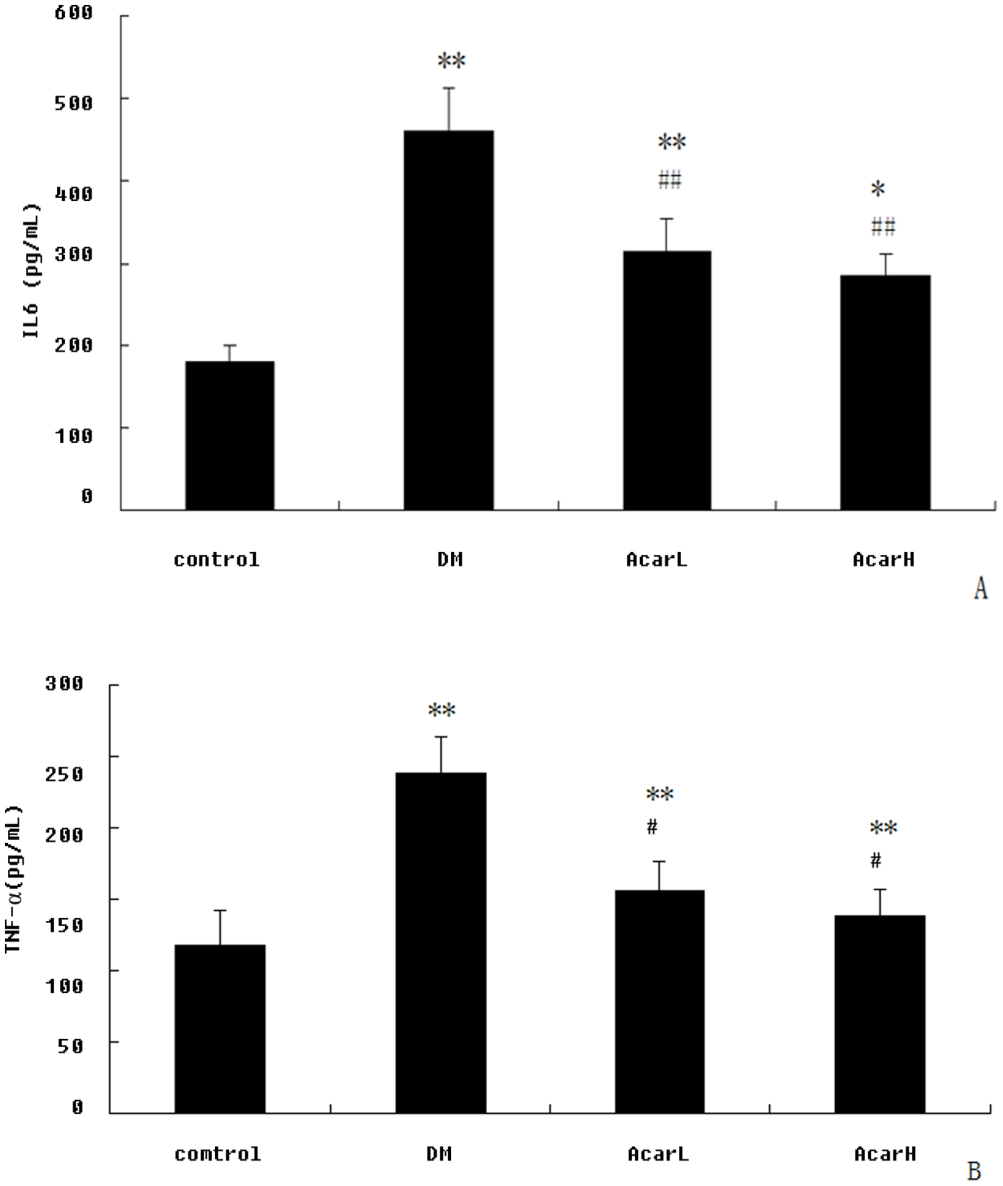
The effect of acarbose on serum IL6 (A) and TNF-α (B) in rats (n = 10 per group). Data represent mean ± SD (n = 10). **P<0.01 versus the control group; ^##^ *P*<0.01 versus DM group.

To determine whether acarbose suppressed inflammatory mediators in DM rats, the levels of serum IL6 and TNF-α were determined. The levels of serum IL6 and TNF-α were significantly elevated in DM rats (*P*<0.01). Acarbose significantly suppressed serum IL6 (*P*<0.05) and TNF-α (*P*<0.01) after the 8-week treatment (Figure 3).

### 5. miRNAs Differentially Regulated by Acarbose

**Figure 4:**
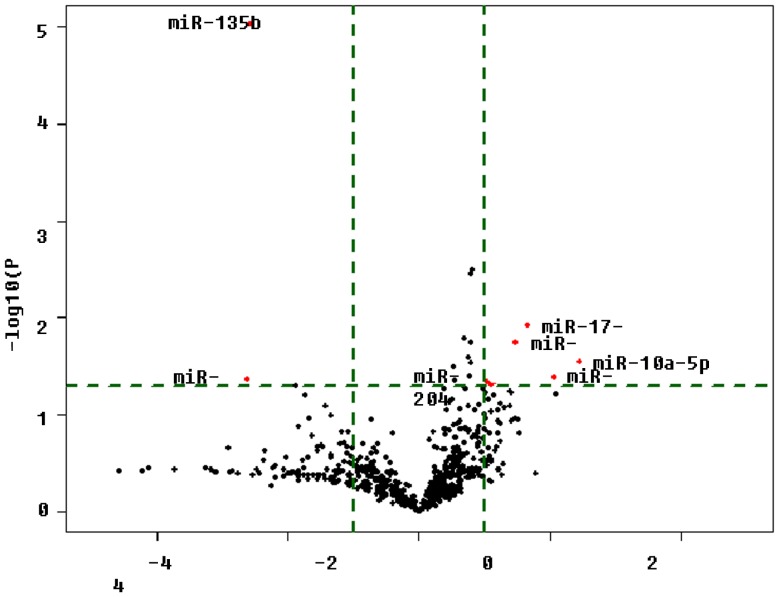
The Volcano Plot graph of miRNA array. This graph shows that log 2 of the fold change in each miRNA’s expression between AcarH group and DM group is versus its -log 10 of *P* value from the *t*-test. The vertical green line indicate that the fold change in miRNA expression threshold is 2. The horizonal green lin indicates that the *P* value of the *t*-test threshold is 0.05. There were 8 miRNAs which showed significantly different expression between AcarH group and DM group.

In the AcarH group, eight miRNAs showed a significant change (fold change>2, *P*<0.05, Figure 1). Both miR-541 and miR-135b were significantly down-regulated. However miR-151*, miR-10a-5p, miR-205, miR-17-5p, miR-145 and miR-664 were up-regulated in the AcarH group (fold change>2, *P*<0.05, Table 2, Figure 4).

**Table 2:** Differentially expressed miRNA (fold change>2, *P*<0.05).

rno miRNA Gene	Fold change	P value	Chromosomal location	Mature sequence
rno-miR-541	0.1619	0.0429	6q32	AAGGGAUUCUGAUGUUGGUCACACU
rno-miR-135b	0.1667	9.93E-06	13q13	UAUGGCUUUUCAUUCCUAUGUGA
rno-miR-151*	4.2461	0.0463	7q34	UCGAGGAGCUCACAGUCUAGU
rno-miR-10a-5p	5.5994	0.0282	10q31	UACCCUGUAGAUCCGAAUUUGUG
rno-miR-205	2.1430	0.0493	13q27	UCCUUCAUUCCACCGGAGUCUGU
rno-miR-17-5p	3.1521	0.0118	15q24	CAAAGUGCUUACAGUGCAGGUAG
rno-miR-145	2.0391	0.0452	18q12.1	GUCCAGUUUUCCCAGGAAUCCCU
rno-miR-664	2.7681	0.0184	18q11	UAUUCAUUUACUCCCCAGCCUA

### 6. miRNA qRT-PCR

**Figure 5:**
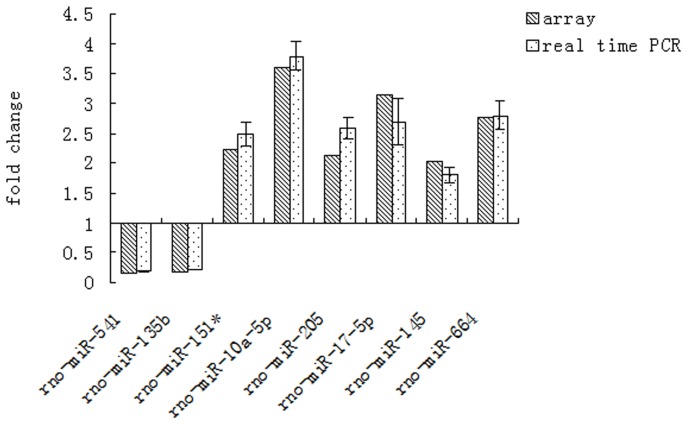
Differential miRNAs expression in gene array and Q-PCR.

To validate the microarray results, all of the miRNAs were selected for qRT-PCR quantification. All of the miRNA expression levels obtained by qRT-PCR were similar to those observed by microarray analysis (Figure 5).

### 7. Identification of the Target Genes of the Differentially Expressed miRNAs Using Bioinformatics Analysis

We next performed bioinformatics analysis to identify the target genes of the differentially expressed miRNAs. Together, the eight miRNAs had 189 validated target genes in the miRWalk database (Table 3). By using the miRWalk and KEGG databases, these 189 validated genes were implicated in the TGF-β signaling pathway, the MAPK signaling pathway, the Wnt signaling pathway and to be involved in the cytokine-cytokine receptor interaction (Table 4).

**Table 3:** Validated targeted genes for defferentially-expressed miRNAs.

miRNA	Target gene
rno-miR-541	*Notch1*
rno-miR-135b	*Mafb*, *Apc*, *Smad5*, *Mybbp1a*, *Stat3*, *Ibsp*, *Mapre1*
rno-miR-151*	*Ptk2*, *Stat5a*, *Prkch*
rno-miR-10a-5p	*Il6*, *Camk2b*, *Casp4*, *Zfhx1b*, *Cd36*, *Zeb1*, *Il10*, *Mafb*, *Map3k7*, *Tnf*, *Ptgs1*, *Syt1*, *Fos*, *Tnfsf10*, *Ccl2*, *Scd2*, *Map3k8*, *Btrc*,*Hoxa7*, *Srsf1*, *Cxcr4*, *Tp53*, *Elavl2*, *Akt1*, *Creb1*
rno-miR-205	*Bad*, *Sox2*, *Zfhx1b*, *Akt1*, *Twist1*, *Apc*, *Notch1T*, *Gadd45a*, *Maf*, *Pten*, *Epb4*, *Sip1*, *Met*, *Egfr*, *Cscl3*, *Pdcd4*, *Pdgfb*, *Rit2*, *Twist2*, *Rad23b*, *Ptgs2*, *Klkb1*, *Mpst*, *Nqol*, *Ptk2*, *Tp53*, *Hif1a*, *Foxa2*, *Yse1*, *Hprt*, *Snai1*, *Stat3*, *Foxp3*, *Nrepps*, *Zeb1*, *Mapk3*, *Cdon*, *Inppl1*, *Kras*, *Bcl212*,*Foxe1*
rno-miR-17-5p	*Apc*, *Bcl12*, *Bcl2l11*, *Cd4*, *Cdkn2a*, *Egln3*, *Foxp1*, *Mtor*, *Kras*, *Myc*, *Mycn*, *Cebpz*, *Pdcd4*, *Plk2*, *Pten*, *Rbl2*, *Ren*, *Runx1*, *Sftpc*, *Smad4*, *Smad5*, *Stat3*, *Tgfbr2*, *Tp53*
rno-miR-145	*Abcb1b*, *Adam17*, *Akt1*, *Bcl2*, *Akt1*, *Bcl2*, *Bitc2*, *Bmp4*, *Camp*, *Cdkn1a*,*Cftr*, *Clock*, *Col1a1*, *Dpagt1*, *Egfr*, *Eif4ebp1*, *Mtor*, *Fcer2a*, *Hoxa9*, *Irs1*, *Itch*, *Kcnh8*, *Klf4*, *Klf5*, *Kras*, *Krt7*, *Bex2*, *Lin28a*, *Mapk3*, *Myc*, *Nfyb*, *Nkx2*, *Sox2*, *Pdgfb*, *Pgk1*, *Ppp3ca*, *Pten*, *Rab27a*, *Atat1*, *Tppp3*, *Serpina1*, *Slc12a2*, *Slc14a1*, *Smad2*, *Smad3*, *Smad4*, *Tagln*, *Tgfbr2*, *Tnfsf10*, *Tp53*, *Fabp4*, *Tsc2*
rno-miR-664	*Acvr1*, *Adora1*, *Akap6*, *Aqp4*, *Capn8*, *Syne1*, *Dclk1*, *Dhcr24*, *Fgf16*, *Fgfr1*, *Gad1*, *Gmfb*, *Gnb1*, *Hmox1*, *Hyou1*, *Itgb1*, *Kcnj16*, *Klf15*, *Madd*, *Mapk1*, *Mapre1*, *Mgst1*, *Mmp9*, *Neurod1*, *Nol3*, *Nr4a1*, *Ogt*, *Ptges*, *Scn3a*, *Slc17a7*, *Stx1a*, *Syt4*, *Tagln*, *Tpm1*, *Vim*, *Vsnl1*

**Table 4:** Validated target genes grouped by KEGG pathway.

KEGG_ID	Term	Count	Genes
rno04350	TGF-beta signaling pathway	13	*BMP4*, *TNF*, *RBL2*, *SMAD7*, *SMAD5*, *TGFBR2*, *SMAD4*, *SMAD3*, *SMAD2*, *MAPK1*, *MAPK3*, *MYC*, *ACVR1*
rno04010	MAPK signaling pathway	18	*EGFR*, *FGFR1*, *TNF*, *PDGFB*, *TGFBR2*, *FGF16*, *TP53*, *NR4A1*, *MAP3K7*, *AKT1*, *MAPK1*, *FOS*, *KRAS*, *MAPK3*, *MAP3K8*, *PPP3CA*, *MYC*, *GADD45A*
rno04310	Wnt signaling pathway	10	*MAP3K7*, *BTRC*, *SMAD4*, *TP53*, *SMAD3*, *CAMK2B*, *SMAD2*, *PPP3CA*, *MYC*, *APC*
rno04060	Cytokine-cytokine receptorinteraction	10	*EGFR*, *IL6*, *TNFSF10*, *TNF*, *CCL2*, *CXCR4*, *MET*, *TGFBR2*, *IL10*, *ACVR1*

### 8. mRNA qRT-PCR

To determine whether local gene expression of inflammatory markers in the ileum were altered by acarbose treatment, the mRNA expression of IL6, Mapk1 and TNF were determined by qRT-PCR. The expression of *Il6*, *Mapk1* and *Tnf* was down-regulated significantly in the AcarH group (*P*<0.05, Table 5).

**Table 5:** Fold change of AcarH vs. DM in gene expression measured by Q-RT-PCR.

Gene symbol	Fold change	*P*_value
*Il6*	−4.3	0.047
*Mapk1*	−3.1	0.020
*Tnf*	−5.2	0.021

*Il6*: interleukin 6; *Mapk1*: mitogen activated protein kinase 1; *Tnf*: tumor necrosis factor.

### 9. Immunohistochemical Staining

**Figure 6:**
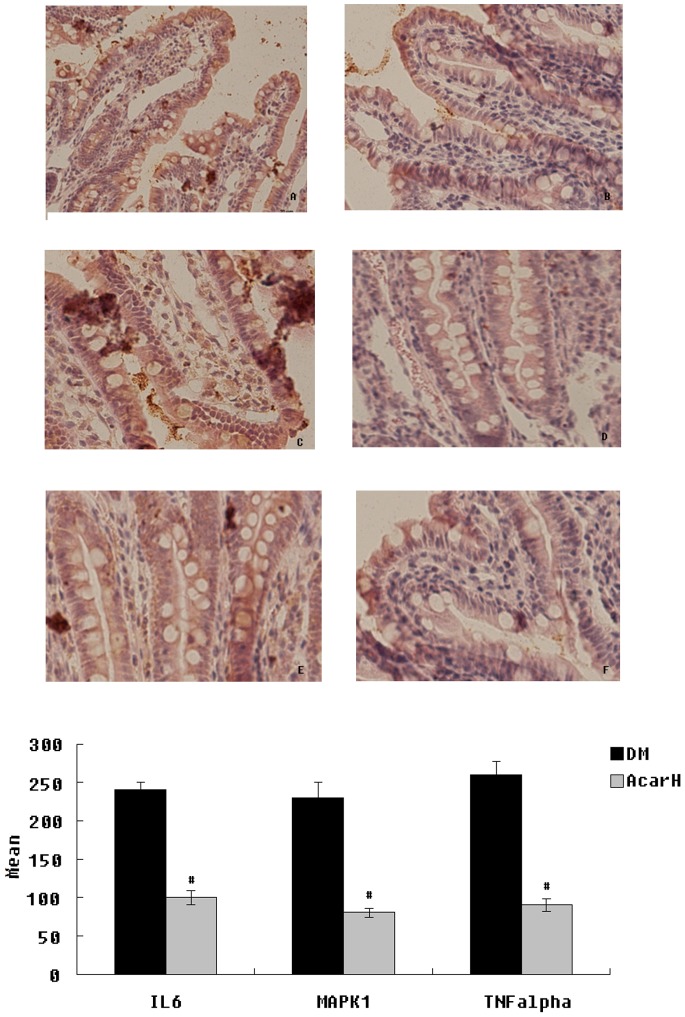
Iluem immunohistochemistry for IL6, MAPK1 and TNF-α expression (original magnification ×200) and semiquantitative assessments. Immunostaining for IL6 (A–B), immunostaining for MAPK1 (C–D), immunostaining for TNF-α (E–F). Iluem were harvested form DM (A, C, E) and AcarH group (B, D, F). Semiquatitatve scores of IL6, MAPK1 and TNF-α (G). Data represent mean ± SD (n = 6). ^#^ *P*<0.05 versus DM group.

To confirm that the protein expression of inflammatory markers were altered in the ileum of DM rats treated with acarbose, immunohistochemistry analyses for TNF-α, IL6 and MAPK1 were performed on ileum tissues. In the AcarH group, there was a statistically significant decrease in the immunoreactivities of TNF-α, IL-6 and MAPK1 (Figure 6).

## Discussion

In this study, the administration of acarbose to diabetic rats significantly reduced blood glucose and altered the expression of miRNAs in the intestine. The miRNAs altered were found to target genes in inflammatory pathways, including *IL6*, *Mapk1* and *Tnf*. In addition, serum IL-6 and TNF-α levels were shown to be significantly decreased in the AcarH group. These results indicate that acarbose reduces inflammation, potentially via miRNA-regulated signals in the intestine. To the best of our knowledge, this is the first report of glucose moderation of acarbose by miRNAs through the inflammatory pathway.

Acarbose is poorly absorbed into the blood stream from the gut. Therefore, we hypothesized that acarbose has a direct effect on the gut to achieve glucose moderation and inflammation reduction. To investigate the effect of acarbose on miRNA expression, potential miRNA targets in the terminal ileum were identified. In our miRNA gene array analysis, eight miRNAs showed significant changes in the AcarH group compared to the DM group. The other miRNAs had 189 target genes that have been experimentally verified. A bioinformatic analysis of all differentially expressed miRNAs in the ileum of the AcarH group, suggested that these genes were involved in the TGF-β signaling pathway, the MAPK signaling pathway, and the Wnt signaling pathway as well as the cytokine-cytokine receptor interaction. qRT-PCR and immunohistochemical staining verified that the expression of *Il6*, *Tnf* and *Mapk1* were down-regulated in the AcarH group. *Il6* and *Tnf* were identified as target genes of miR-10a-5p, and *Mapk1* was one of the target genes of miR-664. These findings suggest that acarbose can reduce the expression of inflammatory cytokines in the ileum through miR-10a-5p and miR-664.

Individuals with type 2 diabetes display the features of low-grade inflammation [16]. Cytokines are a group of pharmacologically active low molecular weight proteins possessing autocrine and paracrine effects [17]. Moreover, they are products and effectors of the inflammatory and immune systems [18]. Chronic hyperglycemia modulates immune responses and triggers inflammation through the activation of Toll-like receptors (TLRs), leading to an increase in the proinflammatory cytokines, IL1, IL6, TNFα and interferon γ(IFNγ), and a decrease in the levels of interleukin 10 (IL10). Proinflammatory cytokines inhibit insulin signals by activating receptor kinase inhibitors, stimulating NFκB to induce pancreatic β cell damage and apoptosis. IL6 can affect glucose homeostasis and metabolism through multiple pathways [19]. IL6 can also increase plasma plasminogen activator inhibiotor (PAI-1) and c-reactive protein (CRP) levels [20] and inhibit glucose-stimulated insulin secretion in islets [21]. Acarbose may upregulate miR-10a-5p to suppress the expression of IL6 in the gut, thereby moderating glucose homeostasis and inflammation in diabetic rats.

TNF-α is another target gene of miR-10a-5p. TNF-α reduces glucose transporter 4 in cells and decreases glucose uptake. TNF-α also impairs insulin action interfering with insulin signaling by binding to TNF-α receptors on muscle cells or hepatocytes [22]. Administering soluble TNF-α receptors in obese rats neutralizes TNF-α and improves insulin sensitivity [23]. Continuous exposure of 3T3-L1 adipocytes to TNF results in a decrease in GLUT4 and insulin receptor (IR) gene expression [24]. Thus, acarbose may moderate glucose metabolism and proinflammation by up-regulating miR-10a-5p and decreasing the expression of TNF-α.

Using the KEGG analysis, we observed that the primary pathway through which miRNAs targeted genes in the acarbose group was the MAPK signaling pathway. qRT-PCR verified this result. MAPK1 was identified as a target gene of miR-664, and TNF-α is interconnected with MAPK pathways. Waetzig *et al.* found that SB 203580, a p38 inhibitor, significantly reduces the secretion of TNF-α in the mucosa [25]. Hollenbach *et al.* found the SB203580 reduces the mRNA levels of proinflammatory cytokines (e.g.,TNF-α, IL2, IL18) in the gut of BALB/c mice [26]. Thus, we propose that acarbose increases the expression of miR-664, leading to decrease Mapk1 expression, which ultimately reduces TNF-α in the gut.

In conclusion, acarbose can regulate glucose metabolism through the MAPK pathway and can suppress of proinflammatory cytokines by increasing miR-10a-5p and miR-664 in the ileum. These results provide molecular information for the future investigation of the mechanisms by which acarbose regulates glucose metabolism in DM rats at the miRNA level. Furthermore, these results could be important in devising mechanism-based and targeted therapeutic strategies for DM. However, future studies using human samples must be carried out to validate our findings and to further elucidate the miRNAs regulatory networks that are affected by acarbose in the treatment of type 2 diabetes.
