# Correction: Acarbose Reduces Blood Glucose by Activating miR-10a-5p and miR-664 in Diabetic Rats

**DOI:** 10.1371/annotation/330eea00-9c09-4982-a458-3add994bab9d

**Published:** 2014-01-21

**Authors:** Qian Zhang, Xinhua Xiao, Ming Li, Wenhui Li, Miao Yu, Huabing Zhang, Zhixin Wang, Hongding Xiang

Errors were introduced during the production process. The placement of some content and text in Figure 4 along the x and y axes is incorrect. The correct version of Figure 4 can be viewed here: 

**Figure:**
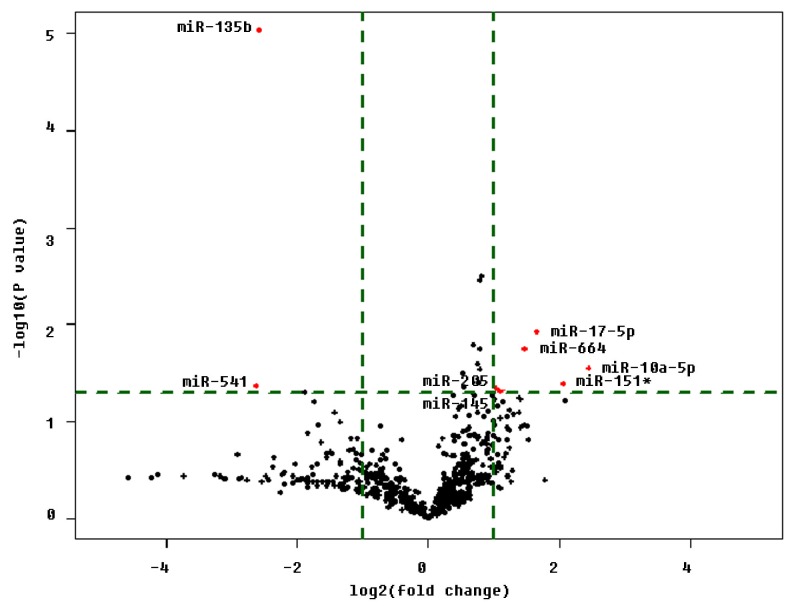


. The publisher apologizes for these errors.

